# Effect of Different Larval Diets on Life History Traits and Nutritional Content in *Anastrepha fraterculus* (Diptera: Tephritidae)

**DOI:** 10.3390/biology14101332

**Published:** 2025-09-27

**Authors:** Fátima L. Fernández, María Josefina Ruiz, Pilar Medina Pereyra, Fabián H. Milla, Alejandra C. Scannapieco, Diego F. Segura, María Teresa Vera, David Nestel, Lucía Goane

**Affiliations:** 1 Facultad de Agronomía, Zootecnia y Veterinaria, Universidad Nacional de Tucumán, San Miguel de Tucumán 4000, Argentina; fatiifernandez96@gmail.com (F.L.F.); josefinaruiz2802@hotmail.com (M.J.R.); m.t.vera@iaea.org (M.T.V.); 2 Consejo Nacional de Investigaciones Científicas y Técnicas (CONICET), Buenos Aires 1425, Argentina; 3 INSUE Instituto Superior de Entomología “Dr. Abraham Willink”, Facultad de Ciencias Naturales e IML e, Universidad Nacional de Tucumán, San Miguel de Tucumán 4000, Argentina; 4 Instituto de Fisiología Animal, Fundación Miguel Lillo, San Miguel de Tucumán 4000, Argentina; mpmedina@lillo.org.ar; 5 Instituto de Genética, GV-IABIMO, INTA-CONICET, Hurlingham 1686, Argentina; milla.fabian@inta.gob.ar (F.H.M.); ascannapieco@agro.uba.ar (A.C.S.); diego.segura@gmail.com (D.F.S.); 6 Departamento de Producción Animal, Facultad de Agronomía, Universidad de Buenos Aires, Buenos Aires 1113, Argentina; 7 Facultad de Ciencias Agrarias y Veterinarias, Universidad del Salvador, Buenos Aires 1020, Argentina; 8 Insect Pest Control Section, Joint FAO/IAEA Centre of Nuclear Techniques in Food and Agriculture, Department of Nuclear Sciences and Applications, International Atomic Energy Agency, 1400 Vienna, Austria; 9 Department of Entomology, Institute of Plant Protection, The Volcani Center, Agricultural Research Organization (ARO), Rishon LeZion 7505101, Israel; nestel@agri.gov.il

**Keywords:** larval nutrition, metabolites content, South American fruit fly

## Abstract

*Anastrepha fraterculus* (Diptera: Tephritidae) is a major fruit pest in several countries of South America and is mass-reared for use in integrated pest control strategies, including the sterile insect technique (SIT), and as a host for rearing biocontrol agents. Optimizing these rearing protocols requires a deeper understanding of how larval diet impacts adult traits. This study investigated the effects of three larval diet formulations differing in nutrient composition on larval development and adult fitness traits. All diets contained inactive non-hydrolyzed brewer’s yeast and sucrose. Two of them included wheat germ, either alone (wheat germ diet) or combined with mashed carrot (carrot diet), whereas the corn flour diet did not contain wheat germ. The carrot diet produced the heaviest pupae, adults with longer wings, and the lowest rate of deformed adults. The corn flour diet prolonged larval and pupal development and increased adult lipid and carbohydrate content. Both the corn flour and carrot diets led to greater glycogen accumulation and more skewed weight distributions compared to the wheat germ diet. Present results highlight how larval diet composition determines developmental traits with direct consequences on adult physiology in *A. fraterculus*. These characteristics could enhance the effectiveness of control programs such as SIT and other biological control strategies.

## Simple Summary

The success of sustainable pest control strategies, such as the sterile insect technique and the use of biological control agents, relies on the mass production of high-quality insects. One of the most influential factors during insect production is the nutrition provided during the larval stage, which can impact adult traits like body size, wing development, and energy reserves of newly emerged individuals. In this study, we evaluated different artificial diets based on widely available ingredients like wheat germ, carrots, and corn flour to determine how larval nutrition affects key adult traits of *Anastrepha fraterculus* (Diptera: Tephritidae). Our results showed that larval diets confer distinct physiological benefits. The carrot with wheat germ diet promoted larger adult size, which may be advantageous for mating competitiveness, while the corn flour diet favors energy storage, potentially enhancing survival under stressful or resource-limited environments. These findings highlight how larval diet can differentially shape developmental outcomes or adult physiology of tephritid fruit flies.

## 1. Introduction

Nutrition is a key determinant of biological fitness in tephritid fruit flies (Diptera: Tephritidae), influencing survival, developmental time, body size, reproductive capacity, and other life history traits such as tolerance to environmental stress [1,2,3,4,5]. Flies incorporate and accumulate metabolic reserves in the larval stage as a result of nutrient assimilation, including key metabolites such as proteins, carbohydrates, and lipids [6]. The relative concentration of these macronutrients significantly affects larval [7] and adult performance, particularly via lipid and protein accumulation during larval development [8,9,10,11]. Consequently, recent efforts have been focused on developing cost-effective artificial larval diets using alternative ingredients, such as corn flour, soybean flour, oatmeal flour, carrot (*Daucus carota* L.), and vegetable oils, aiming to enhance rearing efficiency while maintaining key quality traits of the insects, such as survival, size, reproductive capacity, and mating competitiveness [4,12,13,14,15,16]. In *Anastrepha ludens* (Loew), the inclusion of carrot fiber in the larval diet resulted in increased pupal weight, higher egg production per female, and improved cold tolerance [14]. In contrast, diets supplemented with coconut fiber led to greater egg-to-larva survival and higher pupation rates [14]. Similarly, in *Ceratitis capitata* (Wiedemann), a diet containing sugarcane bagasse enhanced several biological parameters, including the number of larvae and pupae, as well as larval and pupal weight, when compared to corn flour-based diets [17]. These results highlight how specific larval dietary components can differentially influence developmental outcomes and, in turn, adult functional traits of tephritid fruit flies [18,19]. This underscores the need to improve larval diet formulations as a key strategy to enhance mass-rearing efficiency and biological quality, particularly within area-wide control programs that implement the sterile insect technique (SIT) [18,19].

*Anastrepha fraterculus* (Wiedemann), known as the South American fruit fly, is a major polyphagous fruit pest native to South America, ranging from northern Mexico to Argentina [20,21]. This species infests over 100 fruit species, many of economic importance [22,23]. The nominal species corresponds to a cryptic species complex with multiple morphotypes, of which only the Brazilian species 1 (*A. fraterculus* sp. 1) has been recorded in Argentina [24,25]. Significant efforts have been made to understand the biology of this species, and to develop efficient mass rearing techniques for SIT application [13,23,26,27,28]. These efforts have led to the establishment of mass-rearing initiatives, first in Brazil [28,29] and more recently in Argentina [30].

The first artificial larval diet developed specifically for *A. fraterculus* consists of equal proportions of sugar, wheat germ, and non-hydrolyzed brewer’s yeast, with agar serving as the diet matrix [31]. Among these components, wheat germ contributes with a complex mix of vitamins, minerals, proteins, and essential fatty acids, making it a nutritionally rich ingredient [32]. Alternative ingredients to wheat germ are continually being explored to reduce diet costs without compromising insect quality [15,16]. Several studies have tested carbohydrate-rich flours as potential substitutes for wheat germ. For instance, diets formulated with corn flour combined with carrageenan have shown high efficiency in terms of productivity and quality of *A. fraterculus*, enhancing parameters such as the number of larvae and pupae obtained, larval and egg-to-pupa recovery, emergence rate, and flight ability [16]. In turn, rice flour-based diets were found to support adequate biological development and offer similar nutritional profile to wheat germ-based diets, showing similar values for fecundity, pupal weight, and the duration of the egg and pupal stages [15]. Carrot has been evaluated for its contribution to larval development; for example, when used as lyophilized powder it resulted in variable [33] or low [16] egg-to-pupa recovery, whereas boiled or fresh carrot yielded better developmental outcomes [34]. Moreover, as a natural source of provitamin A (β-carotenes) and antioxidant compounds [35,36], carrots may increase pupal weight as evidenced for other insect species [14,37]. However, despite their nutritional potential, carrot-based formulations have not been adopted for mass rearing, mainly because of the high costs of carrot-based ingredients [13].

This study examined the effects of different larval diet formulations, based on carrot with wheat germ, corn flour, or wheat germ, on both larval development and adult traits, such as wing size, body weight, and female fecundity. The study also assessed the metabolite composition of newly emerged adults to gain insight into the metabolic reserves retained through metamorphosis, offering a more comprehensive understanding of how larval nutrition influences adult traits of flies.

## 2. Materials and Methods

### 2.1. Insects

Pupae of *A. fraterculus* were obtained from a laboratory colony maintained at the Instituto de Genética (GV-IABIMO, INTA-CONICET, INTA Castelar, Buenos Aires, Argentina). Groups of 300 pupae were placed in 12 L plastic containers (22 × 30 × 19 cm) covered with Lycra mesh until adult emergence. The emerged flies were provided with unlimited access to water and an artificial diet consisting of a mixture of sucrose and hydrolyzed brewer’s yeast (MP Biomedicals, Solon, OH, USA) in a 3:1 ratio. Once the flies reached sexual maturity and mated, an oviposition substrate was provided for 24 h. The oviposition substrate consisted of 15 cc plastic containers with colored water, covered with Parafilm^®^, and placed on top of the container with the flies. Then, the eggs were transferred, in groups of 50, to a humid chamber, which consisted of black filter paper (1 cm^2^) placed on a moist cloth inside a Petri dish. After 48 h, the filter paper with the eggs was transferred to new Petri dishes containing the larval diets. The 48 h period was selected because it allows embryos to develop without overexposure to the acidic environment of the artificial diets [28].

### 2.2. Larval Diet Composition and Diet Elaboration

Three larval diet formulations were tested (Table 1). All of them included non-hydrolyzed brewer’s yeast (CALSA, Manantial, Tucumán, Argentina) and sucrose (Ledesma, Libertador San Martín, Jujuy, Argentina) but differed in additional ingredients. One diet included wheat germ and agar, and corresponded to the diet developed by Salles [31], hereafter referred to as the wheat germ diet. This was the first diet widely adopted for *A. fraterculus* rearing and is nowadays considered a standard diet. The second diet included corn flour (Presto Pronta, Arcor, Arroyito, Córdoba, Argentina), hereafter referred to as the corn flour diet, and is an adaptation of the *A. fraterculus* diet developed in Brazil [28,29]. The third diet included mashed carrots with wheat germ, hereafter referred to as the carrot diet, and this formulation was designed based on the potential nutritional value of carrots as a natural source combined with the proven nutritional richness of wheat germ.

For the wheat germ diet, agar was dissolved in water and heated to boiling. For the corn flour diet, corn flour was cooked with half of the total water; once the water reached boiling point, the corn flour was gradually added while stirring continuously to prevent the formation of lumps. This step was carried out to gelatinize the starches and facilitate better integration with the remaining ingredients during mixing. For the carrot diet, carrots were cut into small slices and boiled for approximately 10 min at 100 °C. After boiling, they were blended with half of the water (4.54%) using a hand blender (model Liliana AH508 Practicheff, Granadero Baigorria, Santa Fe, Argentina). Each of these mixtures was then combined with the remaining ingredients, which had been previously mixed with water for 5 min. The resulting mixtures were further homogenized using the same hand blender described before until a homogeneous consistency was achieved. Finally, the pH of each diet was measured using a digital pH meter (model EZ-9909-SP, Shenzhen, China) and recorded during three different preparation events, depending on egg availability (see below).

### 2.3. Experimental Procedure

**Figure A1:**
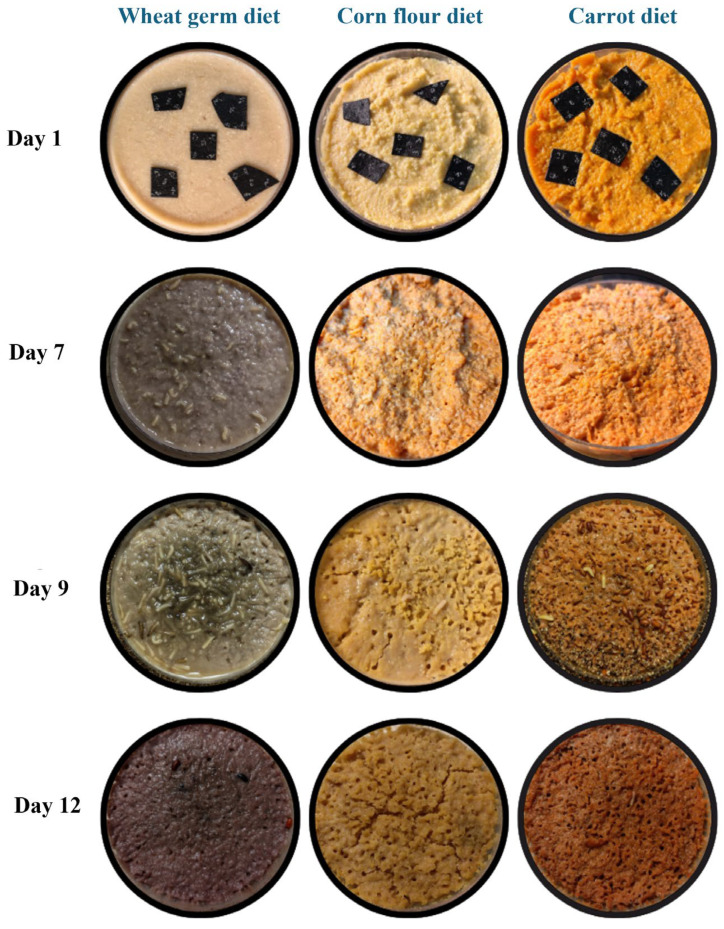
Images of the artificial experimental diets on 10 cm Petri dishes used to rear *Anastrepha fraterculus*.

The experimental unit (larval developmental unit) consisted of a Petri dish (90 mm diameter) containing 60 g of the diet and inoculated with 300 eggs (Figure A1). This larval density was selected to avoid intraspecific competition, based on standard rearing practices for *A. fraterculus* [28]. Three cohorts of eggs were collected on different dates. Within each cohort, multiple larval developmental units depending on egg availability (4 from the first egg collection date, 3 from the second, and 7 from the third) were established. A total of 14 larval developmental units were evaluated per diet. For each cohort, a freshly prepared diet was used. Larval developmental units were incubated at 29 ± 2 °C and 40 ± 5% relative humidity (RH) for the first 24 h to favor egg hatching and then incubated at 25 ± 1 °C and 85–90% RH for the following 7 days to allow larval development. After this time, the filter paper containing the eggs was removed, and each larval developmental unit was transferred into a plastic container with vermiculite for larval pupation. These containers were covered with a voile mesh to prevent infestations by *Drosophila melanogaster* (Meigen, Kirishima, Japan) (Diptera: Drosophilidae). Pupae were daily collected and maintained in vermiculite under controlled conditions (25 ± 1 °C and 60–70% RH) until adult emergence.

#### 2.3.1. Biological Traits

Egg hatch (%), number of pupae, pupal weight (mg), the proportion of larva-to-pupa survival, larval developmental time (days), pupal developmental time (days), the proportion of larva-to-adult survival, the proportion of adult emergence, and sex ratio were estimated for each larval developmental unit. The number of emerged flies was also recorded taking into account whether they were non-deformed, deformed, or partially emerged. Egg hatch (%) was estimated as the number of hatched eggs 5 days after egg seeding/total number of eggs × 100. Pupal weight was estimated by dividing the total weight of pupae (analytical scale, YS202, Ohaus Corporation, Parsippany, NJ, USA) by their number for each larval developmental unit. Pupal weight was measured at four time points, since pupation did not occur simultaneously among larval diets. The larva-to-pupa survival proportion was estimated as the total number of pupae recovered divided by the initial larval density (i.e., the number of hatched eggs). The larval developmental time was estimated as the mean time (days) required for the larvae to pupate. The pupal developmental time was calculated as the mean time (days) required for pupae to emerge as adults. The larva-to-adult survival was calculated as follows: (adult emergence proportion × number of pupae)/number of hatched eggs. The proportion of adult emergence was calculated by dividing the number of emerged adults by the total number of pupae recovered per each larval diet. The proportion of non-deformed, deformed, and partially emerged adults was calculated by dividing the number of individuals in each category by the total number of adults obtained from each larval diet. The sex ratio was estimated as the number of females divided by the total number of males and females. Adult emergence and sex ratio were estimated following the Product Quality Control for Sterile Mass-Reared and Released Tephritid Fruit Flies Manual [38].

#### 2.3.2. Female Fecundity

In addition to assessing the effect of different diets on development, the fecundity of females emerging from each larval diet was compared. Groups of 20 adults (10 females and 10 males) were placed in plastic containers with unlimited access to water and a diet consisting of sucrose and hydrolyzed brewer’s yeast at a 3:1 ratio. A total of five fly groups were evaluated for each larval diet, with each container considered an experimental unit. When flies were 10 days old, oviposition substrates (described in Section 2.1) were provided at the top of the container (covered with a Lycra mesh) for 24 h. This procedure was repeated for six consecutive days (i.e., six different ages of females), and the number of eggs laid by females each day was recorded.

#### 2.3.3. Wing Length

Wing length (mm) was measured in 40 flies (20 females and 20 males) from each larval diet. Measurements were made under a trinocular stereoscopic magnifier (Biotraza XTD-217T, Biotraza, Hangzhou, China) equipped with an MShot MS60 digital camera (BIO-OPTIC, Guangzhou, China). Following Sciurano et al. [39], the length of the right wing of each fly was measured using the MShot Image Analysis System software (v 1.0, Micro-shot Technology Co., Guangzhou, China).

#### 2.3.4. Dry Weight and Metabolite Content

Dry weight (mg) and metabolite content (μg/mg) were measured in newly emerged individuals. A total of 20 newly emerged flies (10 females and 10 males) were randomly selected from each diet and preserved at −20 °C for subsequent quantification of their metabolite content. This procedure was performed on two different dates to ensure flies came from different larval developmental units and egg collections, totaling 40 individuals per diet. Flies were individually dry-weighed using a precision microbalance (0.0001 g, Ohaus Corporation, Parsippany, NJ, USA). Conventional chemical methods were used to quantify nutritional metabolites, as detailed below [40,41,42]. Each individual was homogenized in 180 µL of lysis buffer to solubilize proteins [40] and placed in glass tubes for the quantification of nutritional metabolites. An aliquot of the solution was then extracted and mixed with Coomassie Brilliant Blue G-250 reagent (Biopack^®^, Zarate, Buenos Aires, Argentina) following the Bradford method [41] for protein quantification. To dissolve carbohydrates and precipitate glycogen, a 20% sodium sulfate solution was added prior to centrifugation. Lipids and carbohydrates were determined according to the protocol adapted by Kaufmann [42], which involves successive fractionation steps and heat treatment. Lipids were measured with vanillin reagent (Sigma-Aldrich, St. Louis, MO, USA), while total carbohydrates and glycogen were quantified with anthrone reagent (Cicarelli, Santa Fe, Argentina). Optical density was measured in microplates using an automatic UV/visible spectrophotometer (Thermo Scientific model Multiskan Go, Espoo, Finland). Absorbance readings were taken at 595 nm for proteins, 530 nm for lipids, and 625 nm for carbohydrates and glycogen.

### 2.4. Data Analysis

All analyses were performed in R v.4.4.3 [43].

#### 2.4.1. Biological Traits

Egg hatch (%), the proportion of larva-to-pupa survival, the proportion of larva-to-adult survival, the proportion of adult emergence, and each category of emerged adults were analyzed using generalized linear mixed models (GLMM) with a binomial distribution and a logit link. Since overdispersion was detected, GLMMs with beta-binomial distribution and a log link function were performed (preferred over quasibinomial distribution because a random effect was included [44]) (glmmTMB package) [45]. Number of pupae was analyzed using a GLMM with a Poisson distribution. Since overdispersion was detected, a GLMM with a negative binomial distribution and a log link function was performed. Sex ratio was analyzed using a GLMM with a binomial distribution and a logit link. Pupal weight, larval development time, and pupal development time were analyzed using linear mixed models. The residuals were examined for normality and homoscedasticity. Since these assumptions were not met, GLMMs were applied with a gamma distribution and a log link function. In all models, larval diet was included as a fixed effect and cohort as a random effect. Post hoc comparisons were performed using the emmeans package [46], and significant differences were indicated with letters (multcompView package) [47].

#### 2.4.2. Female Fecundity

Fecundity data (eggs laid by ten females per day) was analyzed using a generalized linear model (GLM) with a Poisson distribution. Since overdispersion was detected, GLM with quasi-Poisson distribution and a log link function was performed. Larval diet, female age, and their interaction were included as fixed effects (stats package) [43].

#### 2.4.3. Wing Length

Wing length was analyzed using a linear model. The residuals were examined for normality and homoscedasticity. Since heteroscedasticity was detected, a GLM with a gaussian distribution was performed. Larval diet, sex, and their interaction were included as fixed effects (glmmTMB package) [45]. Post hoc comparisons were conducted with the emmeans package [46], and significant differences were indicated with letters using the multcompView package [47].

#### 2.4.4. Dry Weight

Dry weight was analyzed using a linear mixed model. Normality and homoscedasticity were confirmed for residuals (stats package) [43]. Larval diet, sex, and their interaction were included as fixed effects, and two different dates as a random effect. Post hoc comparisons were conducted with the emmeans package [46], and significant differences were indicated with letters using the multcompView package [47].

#### 2.4.5. Metabolite Content

Metabolite content (proteins, lipids, glycogen, and carbohydrates) was standardized by individual dry weight (µg/mg) [40,48,49]. Metabolite content was analyzed using linear mixed models. Residuals were examined for normality and homoscedasticity. Since these assumptions were not met, GLMMs were applied with a gamma distribution and a log link function (glmmTMB and lme4 packages) [44,50]. In all models, larval diet, sex, and their interaction were included as fixed effects, and two different dates as a random effect. Post hoc comparisons were conducted with the emmeans package [46], and significant differences were indicated with letters using the multcompView package [47].

Additionally, frequency distributions of pupa weight, wing length, dry weight, and metabolite content were visualized using histograms, separated by larval diet and sex, using the dplyr and ggplot2 packages [51,52]. Finally, the multivariate structure in the nutrient composition of flies reared in different larval diets was explored. A non-metric multidimensional scaling (NMDS) analysis based on Bray–Curtis dissimilarities was performed using the metaMDS function from the vegan package of R [53].

## 3. Results

### 3.1. Biological Traits

**Figure 1:**
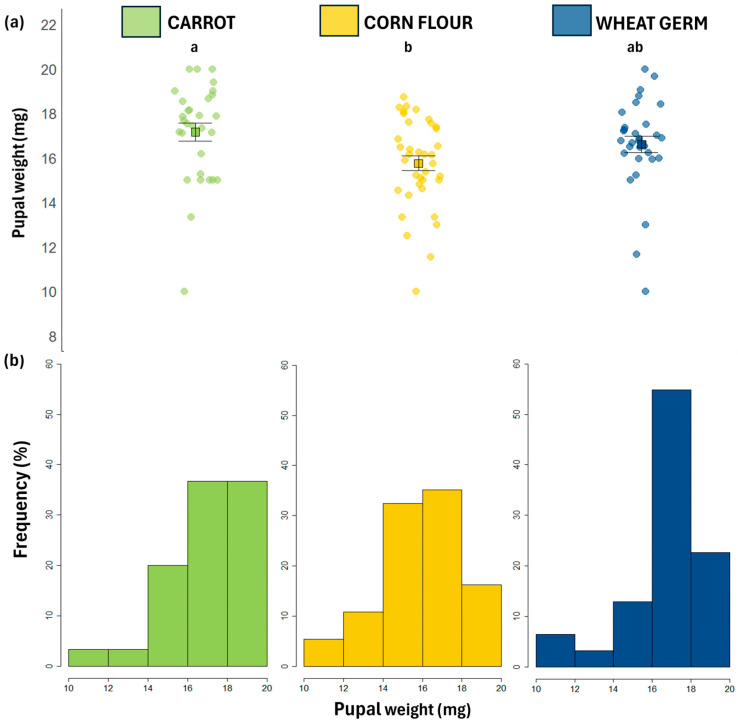
(**a**) Jitter plots showing individual values for pupal weight (mg) of *Anastrepha fraterculus* reared on different larval diets. Black squares represent means, and vertical bars indicate standard errors. Different letters denote statistically significant differences among means based on post hoc Tukey tests (*p* < 0.05). (**b**) Frequency distribution of pupal weights (mg) of *A. fraterculus* reared on different larval diets.

Larval diet affected pupal weight, larval and pupal development times, and the proportion of deformed adults (Table 2). The carrot diet resulted in heavier pupae than those reared on the corn flour diet (Table 2, Figure 1a). This same trend was observed in the frequency distributions, where the carrot diet showed the highest proportion of flies with high weights between 18 and 20 mg (Figure 1b). Larval development time was longer for flies reared on the corn flour diet compared to those reared on the carrot diet (Table 2). Pupal development time was longer for flies reared on the corn flour diet compared to those reared on carrot and wheat germ diets (Table 2). The carrot diet resulted in a significantly higher proportion of non-deformed adults and a lower proportion of deformed adults compared with those emerging from corn flour diet. There was no significant effect of larval diet on the proportion of partially emerged adults (Table 2), as well as on egg hatch (%), number of pupae, larva-to-pupa survival, adult emergence, larva-to-adult survival, and sex ratio (Table 2).

### 3.2. Female Fecundity

Female fecundity was significantly affected by female age (χ^2^ = 10.8533; df = 5; *p* = 0.04939), but not by larval diet (χ^2^ = 2.8711; df = 2; *p* = 0.2379), nor by the interaction between larval diet and female age (χ^2^ = 7.4158; df = 10; *p* = 0.6856). The peak of eggs laid occurred between days 11 and 13 after adult emergence.

### 3.3. Wing Length

**Figure 2:**
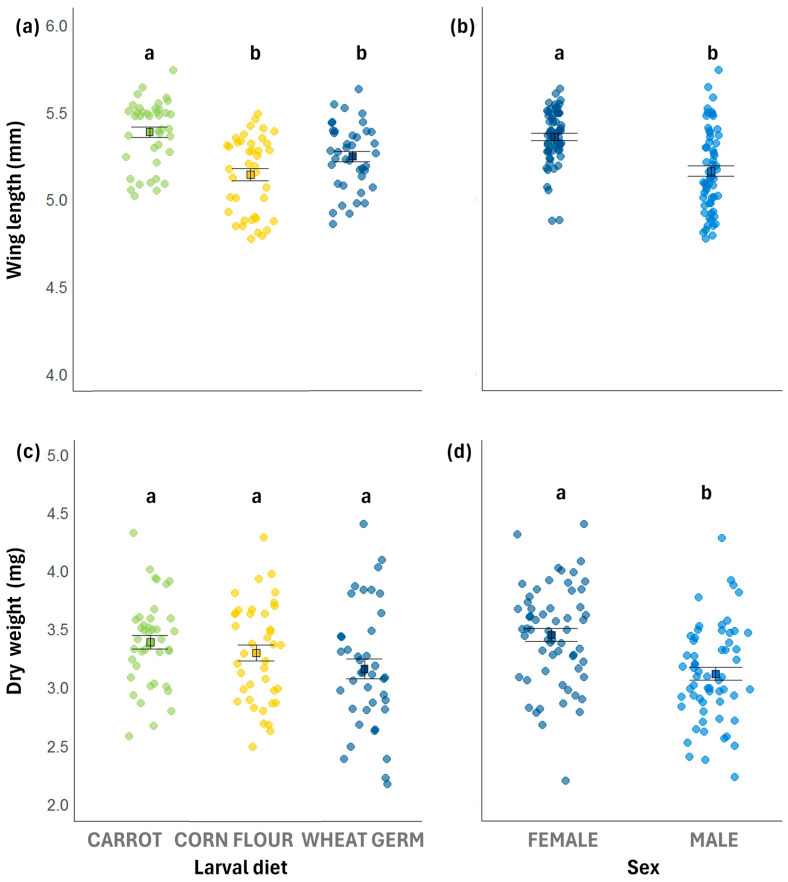
Jitter plots showing individual values for wing length and dry weight of *Anastrepha fraterculus*. (**a**) Wing length (mm) of adults reared on different larval diets; (**b**) wing length (mm) according to sex; (**c**) adult dry weight (mg) reared on different larval diets; (**d**) dry weight (mg) according to sex. Black squares represent means, and vertical bars indicate standard errors. Different letters denote statistically significant differences among means based on post hoc Tukey tests (*p* < 0.05).

**Figure 3:**
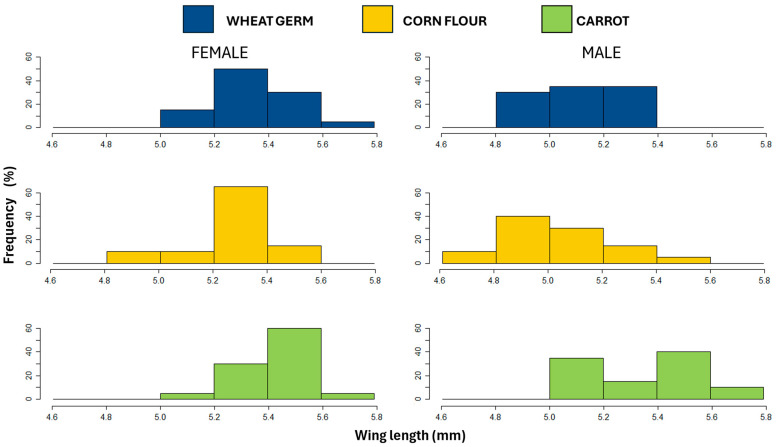
Frequency distribution of wing length (mm) of females and males of *Anastrepha fraterculus* reared on different larval diets.

Wing length was significantly affected by larval diet and sex but not by interaction between larval diet and sex (Table 3). Wing length was greater for flies reared on the carrot diet compared to those reared on the wheat germ and the corn flour diets, which did not differ significantly from each other (Table 3, Figure 2a), with females showing longer wings than males (5.36 ± 0.02 mm and 5.16 ± 0.02 mm, respectively) (Figure 2b). Additionally, the carrot diet resulted in a higher frequency of females and males with long wings than the corn flour or the wheat germ diets (Figure 3).

### 3.4. Dry Weight

Dry weight of adults was significantly affected by sex, but not by larval diet (Figure 2c) or by the interaction between sex and larval diet (Table 3). Females showed higher dry weight than males (3.45 ± 0.05 mg and 3.12 ± 0.05 mg, respectively) (Figure 2d). Additionally, the frequency distribution showed a similar pattern, with high proportions of heavier females compared with males (Figure S1).

### 3.5. Metabolite Content

#### 3.5.1. Glycogen Content

Glycogen content was not significantly affected by larval diet, sex, or the interaction between sex and larval diet (Table 3). However, the wheat germ diet produced a high proportion of flies with low glycogen content (between 0.5 and 2 µg/mg) (Figure S2). The corn flour diet resulted in flies with a broader and more homogeneous distribution (between 0.5 and 5 µg/mg), while for the carrot diet some males reached glycogen levels higher than 6.5 µg/mg (Figure S2).

#### 3.5.2. Lipid Content

**Figure 4:**
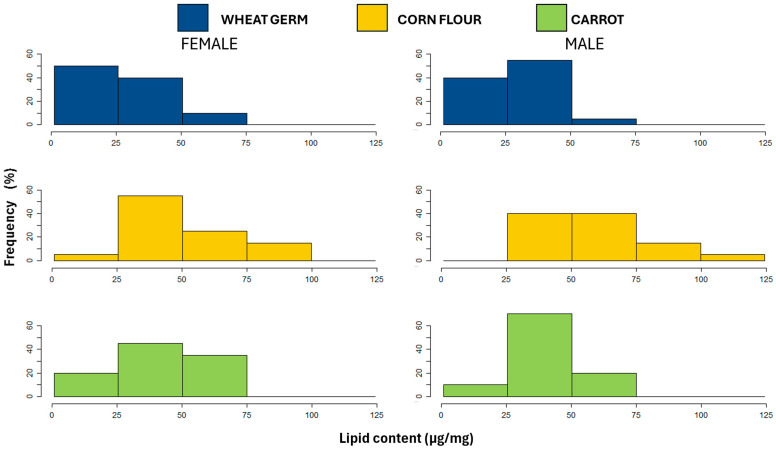
Frequency distribution of lipid content (µg/mg) for females and males of *Anastrepha fraterculus* reared on different larval diets.

Lipid content was significantly affected by larval diet, but not for sex or the interaction between sex and larval diet. Flies reared on the corn flour diet had higher lipid content compared to flies derived from the other larval diets (Table 3). Furthermore, the wheat germ diet produced a high proportion of females and males with low lipid content (between 0 and 75 µg/mg). However, a wide frequency distribution was recorded for females and males reared on corn flour diets (0 to 100 µg/mg and 25 to 125 µg/mg, respectively). The carrot diet produced a high proportion of females with lipid content between 25 and 75 µg/mg, while a high proportion of males between 25 and 50 µg/mg, with no individuals showing extreme values (Figure 4).

#### 3.5.3. Carbohydrate Content

Carbohydrate content was significantly affected by larval diet, but not by sex or the interaction between sex and larval diet. Flies reared on a corn flour diet had higher carbohydrate content than those reared on wheat germ diet (Table 3). On the wheat germ diet, females and males showed a bimodal pattern ranging from 0 to 12 µg/mg, and from 36 to 48 µg/mg, with some individuals reaching values as high as 60 µg/mg of carbohydrates. In contrast, both sexes reared on the corn flour diet were more frequently found in the 12–36 µg/mg range. A comparable pattern was observed in carrot-fed flies, with a high frequency of females and males ranged from 12 to 36 µg/mg (Figure S3).

#### 3.5.4. Protein Content

Protein content was significantly affected by sex, but not by larval diet or the interaction between sex and larval diet (Table 3). Males had a higher protein content (107.74 ± 3.80 µg/mg) than females (99.18 ± 3.50 µg/mg). Protein content showed a comparable frequency distribution across the diets (Figure S4).

#### 3.5.5. Non-Metric Multidimensional Scaling Analysis (NMDS)

**Figure 5:**
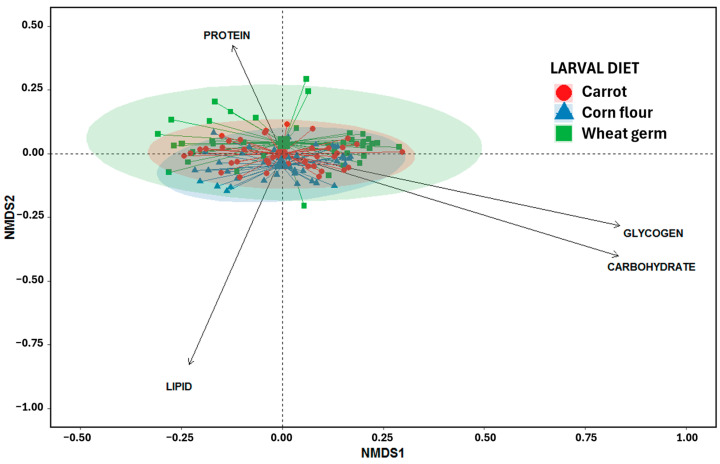
Non-metric multidimensional scaling analysis (NMDS) for metabolite contents of *Anastrepha fraterculus* discriminated against by different rearing larval diets (symbols and colors). Web centers represent the weighted centroids for larval diets. Ellipses group larval diets with a 95% confidence interval.

The NMDS analysis revealed substantial overlap among groups based on larval diet. Flies grown on corn flour and carrot diets exhibited less dispersion in multivariate space compared to those fed the wheat germ diet, indicating greater homogeneity in their metabolite profiles. Notably, flies reared on the corn flour diet clustered more closely along the lipid vector (Figure 5), suggesting a stronger association with lipid-related metabolites.

## 4. Discussion

This study provides insights into how early-life nutrition shapes *A. fraterculus* adult phenotype in the context of artificial rearing techniques. Larval diet composition significantly influenced larval and pupal development times, pupal weight, and wing length, as well as the nutritional metabolite profile of newly emerged adults. The carrot and corn flour diets conferred distinct physiological benefits to flies. The carrot diet resulted in larger adult flies, which may be advantageous for mating success [54], whereas the corn flour diet favored energy storage, potentially enhancing survival under stressful or resource-limited environments [5].

Among the immature traits evaluated, larval and pupal development times, as well as pupal weight, were sensitive to the dietary treatments tested. Flies reared on the corn flour diet (with the highest carbohydrate content, Table A1 in [55]) exhibited significantly prolonged development times, a pattern consistent with previous findings in *A. ludens* and *Bactrocera dorsalis* (Hendel), where high-carbohydrate diets have been shown to delay larval development [7,56]. This effect reflects nutritional imbalance—specifically, high carbohydrate-to-protein ratio (Table A1) [55], which limits the availability of amino acids essential for somatic growth and tissue synthesis [57]. In holometabolous insects, prolonged development under such conditions has been associated with compensatory growth mechanisms that attempt to reach a critical threshold for pupation [2,57,58]. Moreover, even diets with similar protein content may vary in amino acid digestibility and bioavailability [59], a factor that may influence the contrasting development times observed between our study and others using corn flour diets (e.g., 15).

The carrot diet produced the heaviest pupae, followed by the wheat germ diet. This finding aligns with previous research in *A. ludens*, where carrot fiber diets enhanced pupal mass [14], and in *C. capitata*, where diets containing wheat germ improved both pupal weight and developmental efficiency [60]. The effect observed with the carrot diet may be attributed to the use of boiled carrots, which have been reported to increase the moisture content of the diet in similar studies [61], thereby promoting digestion and nutrient assimilation [62], while also introducing biologically active compounds such as carotenoids and antioxidants [36]. These compounds have been shown to reduce oxidative stress, stimulate the immune system, and improve growth in other insects, such as mealworms [37,61,63], supporting their potential role in enhancing pupal weight. The high water content of fresh carrot (87.7–90.1%) [56] has been shown to enhance larval digestion and metabolism [37] and facilitate the access to essential nutrients that affect growth parameters [63]. A high biomass conversion efficiency revealed an effective utilization of a carrot formulation by larvae of *A. ludens* [13]. The nutrients supplied by carrots (e.g., β-carotenoids) probably acted synergistically with wheat germ to improve protein assimilation and reserve accumulation in the larvae, ultimately leading to the development of heavier pupae.

Higher pupal weight does not unequivocally guarantee larger adult sizes, since efficient nutrient conversion and hormonal regulation play a key role in resource allocation to adult structures during metamorphosis [64]. However, wing length, as an indicator of adult body size, was positively affected by the carrot diet in the present study, supporting the positive relationship between pupal weight and adult body size. As in other tephritids, adult size is determined during the final larval instars and early pupation [65]. Larger body size, particularly in males, has been associated with increased mating success [54,66], crucial traits for SIT-based control strategies. Although differences in dry weight were not statistically significant, the same trend observed in wing length was evident, even when analyzing frequency distributions. The carrot diet resulted in a high frequency of males with higher dry weight. Moreover, the carrot diet resulted in a higher proportion of non-deformed adults at emergence and a lower proportion of deformed individuals, a key quality criterion for mass-reared insects as established in SIT [38].

Interestingly, larval diet had no significant effect on female fecundity, suggesting that reproductive output in *A. fraterculus* may be more dependent on the quality of the adult diet than on larval nutritional input [8,27,48]. However, it is also possible that variations in the diets tested were not substantial enough to elicit detectable effects on adult fecundity. Further investigations incorporating traits more sensitive to larval nutritional history such as ovarian maturation rate or the duration of pre-oviposition period would provide a more comprehensive understanding of larval diet effects on female reproductive potential.

Chemical analyses showed that flies from the corn flour diet had significantly higher carbohydrate and lipid reserves, suggesting a more robust energy profile. This result is consistent with the nutritional composition of this diet, which includes 30% corn flour and yeast, ingredients rich in carbohydrate and starches, favoring energy storage. These findings align with previous studies in other fruit fly species where carbohydrate-rich larval diets were associated with increased energy storage [10,11,14,58,67,68]. Carbohydrate-rich diets can enhance lipid synthesis through metabolic pathways such as de novo lipogenesis, a process well-documented in holometabolous insects [68]. These lipid reserves are not only critical for survival under food-limited conditions but also for sustaining adult activity such as reproduction, flight, dispersal, and post-irradiation recovery [69] in SIT applications. Consistent with these findings, the NMDS analysis revealed that flies reared on the corn flour diet had a slight orientation towards lipids. In parallel, glycogen content, a key indicator of short-term energy availability, did not vary across diets. This lack of significant differences may result from the fact that metabolite analysis was performed on the whole insect body, which could have masked subtle variations. The absence of specific tissue-level evaluation, such as of the fat body, or the analysis of hemolymph, particularly to assess trehalose levels [68], may have limited the detection of more precise patterns. However, a high frequency of flies with glycogen content ≤ 2 µg/mg was obtained from the wheat germ diet. This may reflect a potentially limiting rapid-access energy store for adult functions, such as sustained flight or mating, relative to the other diets evaluated. Conversely, flies from the corn flour diet showed intermediate glycogen levels, with most individuals falling between 2 and 4 µg/mg, consistent with its balanced provision of starches and sugar. Interestingly, flies reared on the carrot diet displayed a broader glycogen distribution, particularly among males, which accumulated high levels in some cases. It should be noted that the carrot diet contains a nutritionally rich ingredient such as wheat germ, whose assimilation could be improved by the bioactive compounds present in carrots (rich in fiber, β-carotene, and antioxidants). These bioactive compounds may enhance nutrient assimilation efficiency and influence metabolic partitioning toward glycogen storage. This diet also presented a higher protein content in its nutritional characterization compared to the other diets analyzed (Table A1). Furthermore, the carrot diet included a higher amount of non-hydrolyzed brewer’s yeast in its formulation compared to the wheat germ diet (Table 1). In addition, although all diets included the same protein and sugar sources (non-hydrolyzed brewer’s yeast and sucrose), potential differences in their proportions do not compromise the overall interpretation of the study, as the observed patterns in larval development and adult traits consistently reflect the effects of larval diet composition.

## 5. Conclusions

Taken together, our findings indicate that carrot and corn flour diets confer distinct physiological benefits to *A. fraterculus*. The carrot diet supports larger adult size, which may be advantageous for mating success, while the corn flour diet favors energy storage, potentially enhancing survival under stressful or resource-limited environments [5,68,69]. These results highlight that larval diet composition significantly influences the nutritional phenotype of the adult flies, particularly the accumulation of key energy reserves such as lipids and carbohydrates. These traits may not be mutually exclusive; thus, future efforts should explore optimized formulations that combine the benefits of both diets. Evaluations of long-term effects across generations, as well as performance under environmental stresses such as desiccation, starvation, or thermal extremes, require further research. The impact on adult reproduction requires further research.

## Figures and Tables

**Table 1:** Composition (% by weight) and pH (mean ± SD, n = 3) of each of the artificial diets tested.

Constituent	Wheat Germ Diet	Corn Flour Diet	Carrot with Wheat Germ Diet
Non-hydrolyzed brewer’s yeast	6.04	7	7
Wheat germ	6.04	-	5
Corn flour	-	30	-
Sucrose	6.04	3	5
Sodium benzoate	0.30	0.20	0.20
Nipagin	0.30	0.35	0.20
Citric acid	0.40	0.60	0.90
Agar	0.40	-	-
Mashed carrot	-	-	72.67
Water	80.48	58.85	9.08
pH	4.53 ± 0.06	3.83 ± 0.05	3.76 ± 0.06

**Table 2:** Biological traits (means ± SE) of *Anastrepha fraterculus* reared on different larval diets, and results of statistical inference. Different letters in the same row denote significant differences at the 5% level, df = 2.

Biological Traits	Wheat Germ Diet	Corn Flour Diet	Carrot Diet	Statistical Parameters	
Egg hatch (%)	87.8 ± 1.7 a	87.1 ± 1.8 a	86.2 ± 1.9 a	χ^2^ = 1.3846	*p* = 0.5004
Number of pupae	108 ± 13.5 a	109 ± 13.4 a	122 ± 14.6 a	χ^2^ = 1.0905	*p* = 05797
Pupal weight (mg)	16.50 ± 0.46 ab	15.60 ± 0.44 b	17.00 ± 0.47 a	χ^2^ = 7.2127	*p* = 0.0271
Larva-to-pupa survival (proportion)	0.41 ± 0.04 a	0.43 ± 0.04 a	0.46 ± 0.04 a	χ^2^ = 1.0595	*p* = 0.5888
Larval development time (days)	11.3 ± 0.65 b	12.7 ± 0.73 a	10.3 ± 0.60 c	χ^2^ = 761.99	*p* < 0.0001
Pupal development time (days)	15.9 ± 0.67 b	16.8 ± 0.71 a	15.0 ± 0.63 c	χ^2^ = 839.84	*p* < 0.0001
Larva-to-adult survival (proportion)	0.31 ± 0.05 a	0.30 ± 0.05 a	0.30 ± 0.05 a	χ^2^ = 0.0579	*p* = 0.9714
Adult emergence (proportion)	0.72 ± 0.05 a	0.68 ± 0.05 a	0.68 ± 0.05 a	χ^2^ = 0.4433	*p* = 0.8012
Non-deformed adult emergence (proportion)	0.966 ± 0.014 ab	0.939 ± 0.024 b	0.985 ± 0.007 a	χ^2^ = 17.976	*p* = 0.0001
Deformed adult emergence (proportion)	0.034± 0.014 ab	0.061 ± 0.024 b	0.014 ± 0.007 a	χ^2^ = 17.976	*p* = 0.0001
Partially emerged adults (proportion)	0.003 ± 0.001 a	0.003 ± 0.001 a	0.004 ± 0.001 a	χ^2^ = 0.2532	*p* = 0.8811
Sex ratio (proportion)	0.49 ± 0.01 a	0.48 ± 0.01 a	0.49 ± 0.01 a	χ^2^ = 0.3275	*p* = 0.8490

**Table 3:** Means (±SE) of wing length (mm), dry weight (mg), and metabolite content (μg/mg) of newly emerged *Anastrepha fraterculus* adults reared on different larval diets. Statistical results consider diet, sex, and their interaction. Means followed by the same letter(s) within a row are not significantly different at the 5% level.

				Statistical Parameters		
	Wheat Germ Diet	Corn Flour Diet	Carrot Diet	Larval Diet	Sex	Larval Diet * Sex
Wing length	5.25 ± 0.03 b	5.14 ± 0.03 b	5.39 ± 0.03 a	χ^2^ = 7.9619	χ^2^ = 16.0296	χ^2^ = 5.6060
				*p* = 0.0186	*p* < 0.0001	*p* = 0.0606
Dry weight	3.16 ± 0.06 a	3.30 ± 0.06 a	3.38 ± 0.06 a	*F* = 3.0010	*F* = 19.2490	*F* = 0.6770
				*p* = 0.5370	*p* < 0.0001	*p* = 0.5101
Glycogen	2.35 ± 0.91 a	2.79 ± 1.04 a	2.54 ± 0.98 a	χ^2^ = 5.4336	χ^2^ = 1.7351	χ^2^ = 2.3335
				*p* = 0.0660	*p* = 0.1877	*p* = 0.3113
Lipid	28.14 ± 3.22 c	51.71 ± 5.92 a	39.79 ± 4.55 b	χ^2^ = 45.040	χ^2^ = 0.9660	χ^2^ = 0.4359
				*p* < 0.0001	*p* = 0.3257	*p* = 0.8042
Carbohydrate	18.82 ± 7.08 b	25.76 ± 9.69 a	22.82 ± 8.58 ab	χ^2^ = 12.2979	χ^2^ = 1.0005	χ^2^ = 0.3213
				*p* = 0.0021	*p* = 0.3171	*p* = 0.8516
Protein	102.41 ± 3.98 a	100.37 ± 4.06 a	107.46 ± 4.26 a	χ^2^ = 2.5259	χ^2^ = 5.2669	χ^2^ = 4.2419
				*p* = 0.2828	*p* = 0.0217	*p* = 0.1199

* df =1 for sex; df = 2 for larval diet and the interaction between both factors.

## Data Availability

The original data presented in this study are openly available in the [Institutional Repository of CONICET Digital] at [http://hdl.handle.net/11336/265753 (accessed on 11 July 2025)].

## Supplementary Materials

The following supporting information can be downloaded at https://www.mdpi.com/article/10.3390/biology14101332/s1, Figure S1: dry weight frequency; Figure S2: glycogen frequency; Figure S3: carbohydrate frequency; Figure S4: protein frequency.

## Abbreviations

The following abbreviations are used in this manuscript:

SIT	Sterile insect technique

## Appendix A

**Table A1:** Nutritional characterization of the larval diet (values per 100 g of diet). Nutritional values for corn flour and carrot were obtained from the United States Department of Agriculture (USDA) National Nutrient Database for Standard Reference (https://fdc.nal.usda.gov/), whereas values for brewer’s yeast and wheat germ were taken from the information provided on the commercial product labels.

Larval Diet	Carbohydrates (g)	Proteins (g)	Lipids (g)
Wheat germ	10.59	4.42	0.55
Corn flour	26.98	5.08	0.33
Carrot	16.93	5.21	0.71
