# Supplementary material for: Effect of Different Larval Diets on Life History Traits and Nutritional Content in Anastrepha fraterculus (Diptera: Tephritidae)
Source: Biology (Basel). 2025 Sep 27;14(10):1332. doi: 10.3390/biology14101332 (PMC12561348; doi:10.3390/biology14101332)

Supplementary Materials

Dry weight frequency

Figure S1. Frequency distribution of dry weight (mg) for females and males of *Anastrepha fraterculus* reared on different larval diets.

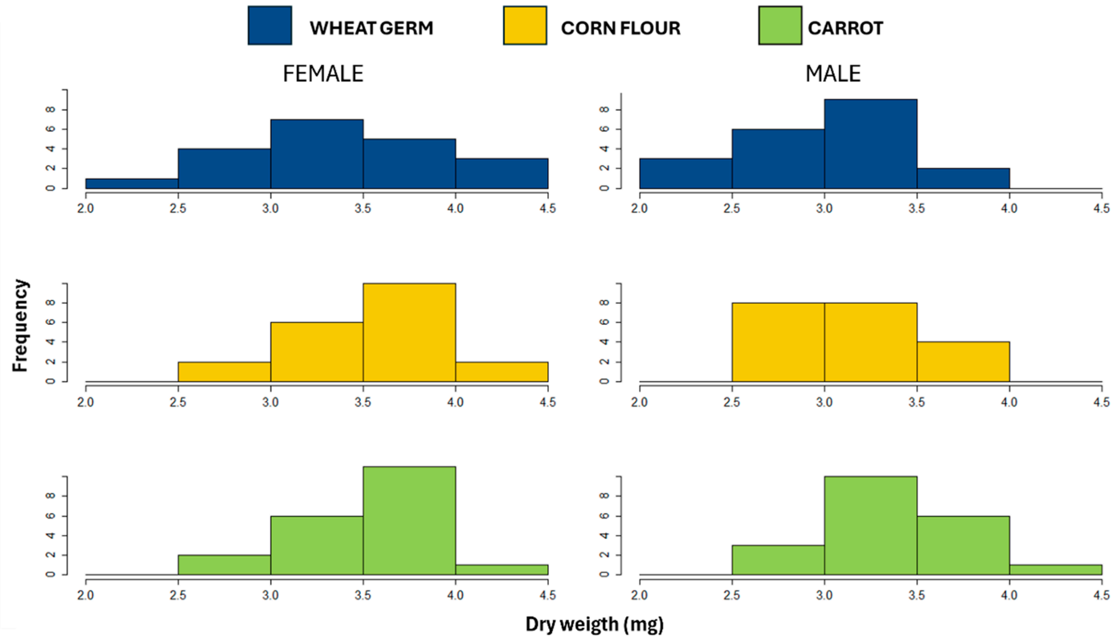

Glycogen frequency

Figure S2. Frequency distribution of glycogen content (µg/mg) for females and males of *Anastrepha fraterculus* reared on different larval diets.

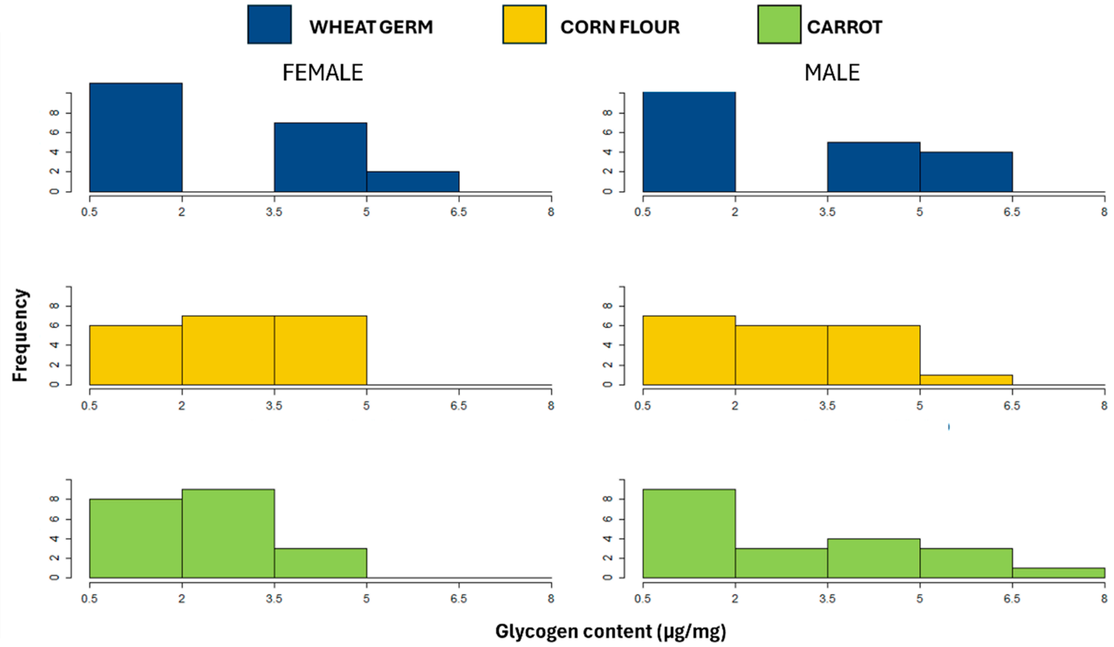

Carbohydrate frequency

Figure S3. Absolute frequency distribution of carbohydrate content (µg/mg) for females and males of *Anastrepha fraterculus* reared on different larval diets.

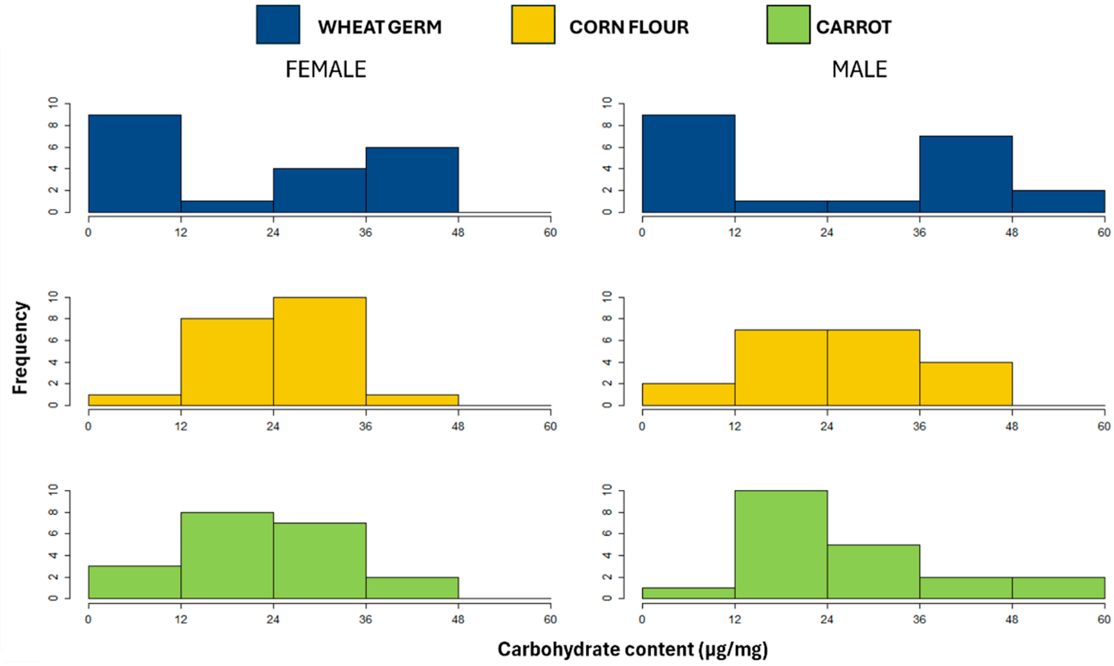

Protein frequency

Figure S4. Frequency distribution of protein content (µg/mg) for females and males of *Anastrepha fraterculus* reared on different larval diets.

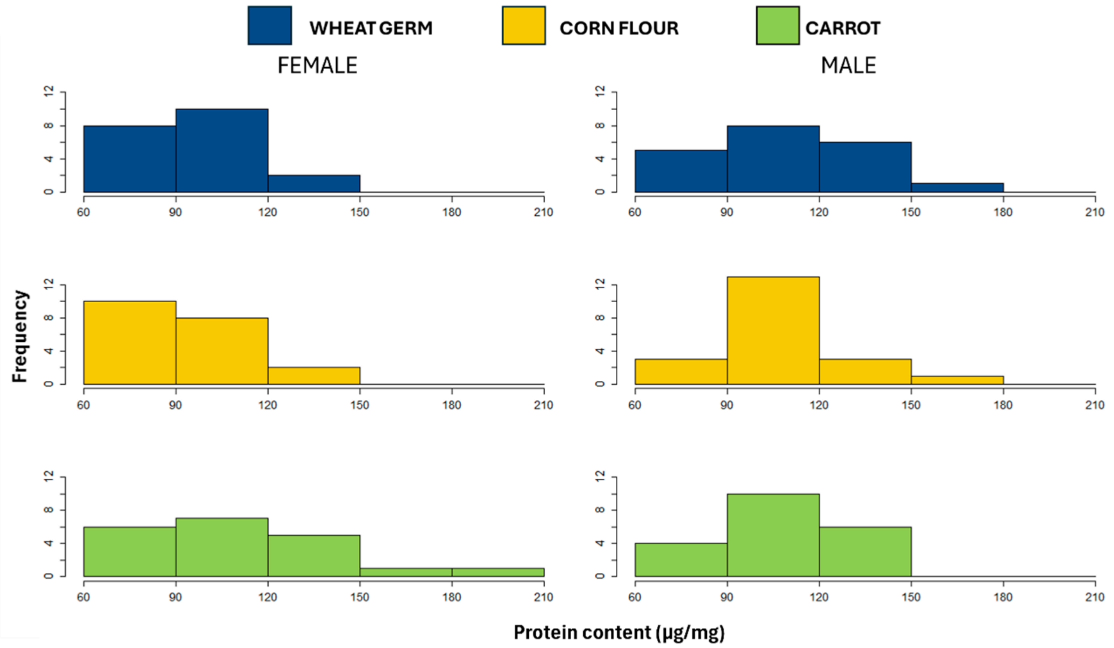

Supplement: Supplementary file 1 [file biology-14-01332-s001.zip › biology-3769927-supplementary.pdf]
